# The effect of the presence of children on adult smoking behaviour: empirical evidence based on China family panel studies

**DOI:** 10.1186/s12889-020-09543-2

**Published:** 2020-09-24

**Authors:** Haoxiang Lin, Chun Chang, Zhao Liu, Huaqing Tan

**Affiliations:** 1 Department of Social Medicine and Health Education, School of Public Health, Peking University Health Science Center, 38. Xueyuan Rd, Haidian District, Beijing, China; 2 Tobacco Medicine and Tobacco Cessation Center, China-Japan Friendship Hospital, No.2 Yinghuadongjie, Chaoyang District, Beijing, China; 3 Guanghua School of Management, Peking University, Beijing, China

## Abstract

**Background:**

Despite a number of studies linking family and marriage factors with health behaviour, the effects of children on the health behaviour of parents are still understudied. This study explored the association between the presence of children and adults’ smoking behaviours.

**Methods:**

This study used panel data from the China Family Panel Studies 2010 and 2012, and the data set included 23,157 households and 45,513 adults. Logistic regression was performed to analyse the association of the presence of children on adults’ smoking behaviours. Subgroup regression was used to examine heterogeneous effects.

**Results:**

Full sample regressions showed that the number of children was significantly inversely associated with smoking behaviour (OR = 0.93; 95% 0.90–0.96). Further subsample regression finds that such effect is only significant among the high-education group (OR = 0.92; 95% 0.87–0.97), high-skill workers (OR = 0.89; 95% 0.80–0.99) and couples who had an age gap greater than 2 years (OR = 0.91; 95% 0.88–0.95).

**Conclusions:**

Our findings confirm the existence of the upward intergenerational effect of the presence of children on adults’ smoking behaviour in China. However, such effects are not equal across all demographic characteristics. Future research could explore other parts of the upward mechanism and possible pathways for a stronger effect. In resource-poor areas, targeting cessation activities at those who have children at an early age may be an effective strategy.

## Background

Tobacco use is a global problem with serious consequences for public health and causes a huge burden on the health system, especially in low- and middle-income countries [1]. The 2015 China Adult Tobacco Survey shows that China has the largest number of smokers worldwide; 52.1% of adult males and 2.7% of adult females are smokers [2, 3].

Social psychological, biological, economic, policy and legislation factors are important determinants of health behaviour. Among all the social psychological characteristics, previous studies mainly focused on marriage, family, education and stress in life [4, 5]. Many studies have indicated that marriage has a positive influence on family members’ health. Some studies have found that compared with people who were divorced or remained single, married people have a lower death rate [6, 7]. Furthermore, Umberson found that marriage might benefit parents’ health by establishing healthy behaviours because family relationships in marriage can provide external regulation and promote self-regulation regarding health behaviours [8, 9].

Despite a number of studies linking family and marriage factors with health behaviour, the effects of children on the health behaviour of parents are still understudied. In particular, reports on the association between the presence of children and adult smoking behaviour are lacking. Several studies have reported some evidence on this topic. Researchers believe that social integration via family status may affect smoking behaviour, and the presence of children and parenting could promote smoking cessation [10]. A study by Bahraini found that, compared to other family members, the presence of children in the home is associated with a lower smoking probability among smokers [11]. One study conducted in Taiwan found that the presence of children under the age of 2 had highly significant effects on reducing the probability of smoking [12].

Because of the sustained increase in life expectancy in the past half century in Mainland China and due to reforms in the country’s family planning policies whereby couples are allowed to have a second child if either parent is an only child [13, 14], the effects of the presence of children on parents’ health behaviour has become increasingly important for both policy makers and scholars. Recently, the association between child factors and parents’ health behaviour was investigated by a cross-sectional Chinese study [15]. However, the upward intergenerational effect of the presence of children on parents’ smoking behaviour has not been studied.

The main hypothesis of the present study was that the presence of children in the household has an inverse association with adults’ smoking behaviour (first hypothesis). Additionally, we explored if the association varied among population sub groups (second hypothesis). This study uses data from the China Family Panel Studies (CFPS) to conduct logistic regressions with fixed effects to explore how the presence of children is associated with adults’ smoking behaviours.

## Methods

### Design and settings

This is a secondary analysis of CFPS data from 2010 and 2012. The CFPS is an ongoing survey designed to collect individual-, family-, and community-level longitudinal data in contemporary China. It was launched in 2010 by the Institute of Social Science Survey (ISSS) of Peking University. The studies focus on the economic-related and non-economic-related well-being of the Chinese population, with a wealth of information covering topics such as economic activities, education outcomes, family dynamics and relationships, migration and health [16, 17].

### Sample and sampling

The CFPS covers 25 provinces and represents 94.5% of the total population in Mainland China. The CFPS uses multistage probability proportional to size sampling (PPS) with implicit stratification to reduce the operational cost of the survey and better represent Chinese society. All the samples were selected through three stages: the primary sampling unit (PSU) was either an administrative district (in urban areas) or a county (in rural areas), the second-stage sampling unit was either a neighbourhood community (in urban areas) or an administrative village (in rural areas), and the third-stage (final) sampling unit was the household. Administrative units and measures of socioeconomic development were used as the main stratification variables.

In 2010, the CFPS successfully interviewed 14,960 households from 635 communities, including 33,600 adults and 8990 children. In 2012, the CFPS successfully followed up with 12,725 of the original households and another 728 new households, including 7185 children below age 16 and another 1439 children who became new members of the sampled households.

To evaluate the extent to which the sample is representative of the population, scholars compared the age–sex structure using the CFPS 2010 data (before weighting) and the census 2010 data. They found that the shapes of the age-sex structure of the two pyramids are almost identical [17].

### Data retrieval

This study used publicly available CFPS data sets downloaded from the CFPS website (https://opendata.pku.edu.cn/dataverse/CFPS). We used the panel data collected in 2010 and 2012 for analysis. The samples we selected were as follows: (1) aged 15–64 years old; (2) reported complete information about smoking behavior, age, marriage and education information in each wave; and (3) reported complete information about whether they had children. We combined the samples we selected from the two data sets.

### Measures

#### Smoking

Respondents were asked to confirm their smoking status from two categories (1 = current smoker in this month, 2 = others). Respondents who were categorized as 1 were then asked when they began smoking and reported more details about their smoking behaviour. Respondents who were categorized as 2 were then asked whether they were ex-smokers or non-smokers.

#### Education

Regarding educational background, respondents were classified as low-education if their highest level of education was middle school (or lower). Respondents were classified as high-education if they obtained at least a high school degree.

#### Number of children

The number of children was calculated by counting how many children an adult had. In our sample, approximately 6.55% of adults do not have children. Approximately 41.54% of adults have one child, and the remaining respondents have two or more children.

#### Occupation

Regarding occupations, the participants were grouped into eight categories: (1) managers or leaders, including the leaders of parties, governments, and companies; (2) specialists, including professionals and technicians; (3) clerks; (4) service workers; (5) producers, including agriculture, forestry, husbandry, and fishery workers; (6) production workers; (7) soldiers; and (8) others. We further classified all respondents into two categories: high-skill workers (chose (1) or (2)) and low-skill workers (chose others).

We excluded soldiers in all samples for two reasons: 1.There are so many branches in the army; some are skilled, and some are not. 2. Soldiers are not free to choose their own occupation. People with high skill may perform low-skill jobs.

#### Age gap

We defined the age gap as the age difference between the husband and his wife. The mean age difference between husbands and wives was 1.89 years in the 2010 CFPS. However, the age difference increased to 3.86 years in the 2012 CFPS. The median age gap for all samples was 2 years. Therefore, we classified the participants by median age gap and performed the regression accordingly: (1) age gap less than or equal to 2 years (excluding couples with younger husbands); (2) age gap more than 2 years. Table 1 presents the definitions of the key variables.

**Table 1:** The definitions of the key variables

Variable	Definition	Mean	Std.	Min	Max
Smoker	If respondents smoked in last month = 1; otherwise = 0	0.289	0.456	0	1
Married	If respondents are married or cohabitating with others = 1; otherwise = 0	0.833	0.373	0	1
Number of children	Number of children in a family	1.231	2.856	0	9
Age gap	The age difference between couple	2.09	3.31	−26	29
Years of education	Years of education	6.73	4.86	0	22
Age	The age of respondent in years	46.591	15.796	16	110

### Statistical analysis

Logistic regression was performed to analyse the association of the presence of children on parents’ smoking behaviours. The specification of our empirical model is as follows:

$$ {y}_{it}={\beta}_0+{\beta}_1\ast {Family}_{it}+{\beta}_2\ast {X}_{it}+{\theta}_i+{y}_t+{\varepsilon}_{it} $$

The dependent variable is a dummy variable indicating whether the respondent was a smoker or not in the last month (if yes = 1; otherwise = 0). The explanatory variable *Family*_*it*_ is the number of children in a family. *x*_*it*_ represent other time-variation control variables, including years of education, marriage and age. We defined age difference as the husband’s age minus the wife’s age. *θ*_*i*_ is the family-level fixed effect, *γ*_*t*_ is the time effect, and *ε*_*it*_ is the error term.

To ensure the consistency of the estimation results, we used the panel data fixed effects model. Therefore, family variables and some time-invariant dummy variables can be controlled and do not change with time. We first regressed the dependent variable on the number of children with other covariates. Then, we divided our sample into several groups based on education level, occupation, age gap and urban vs rural residence to examine heterogeneous effects.

We used STATA 13.1 (Stata Corporation, College Station, TX, USA) to conduct regression analyses. All statistical tests were two-sided, and *P* < 0.05 was statistically significant. CFPS data can be downloaded in DTA format and are available for Stata.

## Result

This study included 45,513 Chinese adults from 23,157 households. Table 2 reports the results of descriptive statistics. Approximately 33.1% of the respondents were in their 50s or older. A total of 62.1% of respondents had a middle school or lower educational level. More than 93.4% of respondents had children, and 16.8% of respondents had more than two children. A total of 28.9% of respondents were current smokers.

**Table 2:** Sample characteristics

Variable	n	%
**Age**		
15–29	9930	21.82
30–39	9010	19.8
40–49	11,510	25.29
50 or older	15,063	33.1
**Gender**		
Male	21,773	47.84
Female	23,740	52.16
**Education attainment**		
Middle school or lower	28,272	62.12
High school or higher	17,241	37.88
**Number of children**		
No children	2981	6.55
have one child	18,906	41.54
have two children	15,953	35.05
More than two children	7673	16.86
**Marriage**		
Married	38,003	83.5
Unmarried (including divorced)	7510	16.5
**Current smoker**		
Yes	13,162	28.92
No	32,351	71.08
**Total**	45,513	100

### Full sample regression to test the first hypothesis

Table 3 presents all sample regressions and estimates the relationship between the number of children and smoking behaviour. The number of children was significantly inversely associated with smoking behaviour (OR = 0.93; 95% 0.90–0.96), and education level was also significantly inversely associated with smoking behaviour (OR = 0.95; 95% 0.92–0.99).

**Table 3:** Estimates of panel regression for presence of children on smoking behavior: full sample

	(1)			(2)		
VARIABLS	Smoke			Smoke		
	Odds Ratio	95% CI		Odds Ratio	95% CI	
Number of children	0.93***	0.90	0.96	0.93***	0.90	0.96
Age				1.02	0.75	1.41
Married				1.08	0.69	1.70
Years of edu				0.95**	0.92	0.99
Year effect	0.53***	0.48	0.59	0.51**	0.27	0.97
Constant	0.93***	0.90	0.95	0.93***	0.90	0.96
Observations	45,513			45,513		
Number of household	23,157			23,157		

Note: *** *p* < 0.01, ** *p* < 0.05;

Model 1 was basic regression without other covariates except the year effect

Model 2 was adjusted for age, married and education based on model 1

### Subgroup analysis by demographic characteristics to test the second hypothesis

To examine differences due to demographic data, we re-conducted the regressions above with subsamples, and the results are shown in Table 4. Table 4A shows whether the association between the number of children and smoking behaviour varied depending on educational level. The results show that such effects are only significant in the high-education group (OR = 0.92; 95% 0.87–0.97). The pattern is similar if we separate the sample by occupation (Table 4B). We only found a significant association in the high-skill workers (OR = 0.89; 95% 0.80–0.99). We also recategorized respondents by whether they were living in urban areas. Table 4C shows the results. We found that the number of children was significantly inversely associated with smoking behaviour in both urban and rural areas.

**Table 4:** Estimates of panel regression for presence of children on smoking behavior: subsamples

**Subsamples regression A: by education attainment**						
	(1)			(2)		
VARIABLES	Smoke			Smoke		
	Middle School and lower			High School and above		
	Odds Ratio	95% CI		Odds Ratio	95% CI	
number of children	1.02	0.96	1.07	0.92***	0.87	0.97
Age	0.37**	0.17	0.79	1.08	0.51	2.28
Married	1.54	0.68	3.52	0.76	0.33	1.77
Years edu	0.93***	0.88	0.98	0.98	0.86	1.13
Year effect	3.73*	0.81	17.17	0.68	0.15	3.00
Observations	28,124			17,389		
Number of household	17,960			12,382		
**Subsamples regression B: by occupations**						
	(1)			(2)		
VARIABLES	Smoke			Smoke		
	High Skilled labor			Low Skilled labor		
	Odds Ratio	95% CI		Odds Ratio	95% CI	
number of children	0.89**	0.80	0.99	1.02	0.94	1.12
Age	0.26	0.05	1.44	1.29	0.61	2.72
Married	0.21*	0.04	1.20	4.35**	1.24	15.27
Years edu	0.96	0.84	1.09	0.91*	0.83	1.00
Year effect	15.51	0.54	448.13	0.27*	0.06	1.18
Observations	8542			17,945		
Number of household	6049			12,801		
**Subsamples regression C: by urban and rural**						
	(1)			(2)		
VARIABLS	Smoke			Smoke		
	Rural			Urban		
	Odds Ratio	95% CI		Odds Ratio	95% CI	
number of children	0.93***	0.90	0.97	0.92***	0.88	0.97
Age	0.78	0.51	1.19	1.30	0.78	2.16
Married	1.08	0.61	1.90	1.14	0.52	2.47
Years edu	0.96*	0.91	1.00	0.93**	0.87	0.99
Year effect	0.73	0.32	1.68	0.43	0.16	1.20
Observations	25,905			19,570		
Number of household	13,175			9978		
**Subsamples regression D: by age gap**						
	(1)			(2)		
VARIABLES	Smoke			Smoke		
	age gap > 2			age gap<=2		
	Odds Ratio	95% CI		Odds Ratio	95% CI	
number of children	0.91***	0.88	0.95	0.96	0.88	1.06
Age	1.09	0.63	1.89	0.20**	0.05	0.72
Married	0.77	0.41	1.44	2.22	0.42	11.78
Years of edu	0.96	0.91	1.01	0.99	0.93	1.07
Year effect	0.42	0.14	1.26	17.73**	1.36	230.37
Observations	10,548			9432		
Number of household	5609			4985		

Note: *** *p* < 0.01, ** *p* < 0.05, * *p* < 0.1

Finally, we investigated the role of age differences in the relationship between the number of children and smoking behaviour. Table 4D shows the results. We only observed that this relationship was significant among couples who had an age gap greater than 2 years (OR = 0.91; 95% 0.88–0.95).

## Discussion

This study contributes the recent debate on whether there exists an intergenerational influence of children on the health of parents, specifically examining the association of having children on smoking behaviour in the Chinese population. This is one of the first studies to use national representative data to test such effects.

As we have proposed, this study confirmed that the presence of children indeed has an inverse association with adults smoking behaviour. This finding is consistent with the findings of Takagi et al. [10], who examined Japanese participants. There are several possible explanations for this association. First, if parents understand that smoking is harmful, they may regulate their smoking behaviour in an effort to improve the health of their children. Second, spouses may monitor and control their partner’s health behaviours when they have children. This is particularly true in China, as the female smoking prevalence is very low [2]. Women are more likely to interfere with their husbands’ smoking behaviour. Third, children and teenagers are the priority of health promotion efforts, and well-educated children might advise their parents to quit.

However, such a effect was only significant in the high-education group and in the high-skill workers. Other studies also found that more educated individuals are more likely to be motivated to protect their health. One possible explanation for these findings is based on education as a factor in the health production function. Scholars found that education can promote access to health-related information and the processing of that information to make health-related decisions [18–20]. This finding indicated that health education programmes should not only consider age or gender but also take into account educational background and occupation. The programme should involve both members of a couple, especially couples with lower educational levels.

Some studies focused on the recent trends in spousal age differences. These studies found that with increasing female educational attainment, women tend to find partners with higher socioeconomic statuses by realizing the pursuit of a better life. On the other hand, men with higher socioeconomic status also tend to find younger and more attractive women [21]. Therefore, among couples with greater age gaps, the percentage of husbands that have better educational attainment or high-skill occupations tends to be higher than among couple with smaller age gaps. These husbands may be more susceptible to positive advice from their partner, especially in China, where the wife usually advise the husband to quit smoking. This could be an explanation for why the couples with a greater age gap experienced a more positive effect from the presence of children.

Our findings have practical implications. In addition to developing comprehensive tobacco control campaigns, policy-makers should pay more attention to the social influences on an individual’s decision to smoke or to stop smoking. According to the 2015 China Adult Tobacco Survey, the majority of smokers had not attempted to quit smoking (only 17.6% smokers wanted to quit smoking within a year). Taking advantage of ‘teachable moments’ to support smokers in quitting is a well-recognized approach to cessation. The presence of children, especially children at an early age, provides opportunities for such ‘teachable moments’ to support smoking cessation. In China, services to help people stop smoking are rare [22, 23]. If limited resources for cessation are to be used effectively, then taking advantage of these ‘teachable moments’ becomes a necessity. Targeting cessation activities at those who have children at an early age is one such strategy.

Furthermore, our study supports the existence of an upward generational effect on adults’ smoking behaviour. When do such effects become effective? How does it influence other health behaviours? All these questions have strong policy implications. Further studies could explore these parts of the mechanism.

There are several limitations in this study. First, the smoking status was based upon self-reported data, without any biomarker validation. However, a study in South Africa confirmed that self-reports are a reliable measure of smoking status [24]. Second, we did not examine the characteristics of the children. It is possible that children’s characteristics explain changes in behaviour rather than the mere presence of children, and we plan to conduct related research in the future. Third, the multivariate analysis was adjusted by demographic factors, but we did not consider physical status or any disease as confounding factors that might have potential influence on their smoking behaviour. Fourth, the data we used are from the years of 2010 and 2012. It is possible that a study including recent data might lead to different results. Fifth, because of CFPS lack some variables that literature shown to be associated with smoking behavior, i.e. peer influence and health-related knowledge etc. hence their association was not tested.

## Conclusion

Our findings confirm the existence of the upward intergenerational effect of the presence of children on parents’ smoking behaviour in China. However, such effects are not equal across demographic characteristics. Future research could explore other parts of the upward mechanism and possible pathways for a stronger effect. In resource-poor areas, targeting cessation activities at those who have children at an early age may be an effective strategy. Taken together, the results of this empirical analysis not only contribute to identifying the determinants of the smoking behaviour of people in Mainland China but also provide further evidence regarding smoking behaviour in a developing country, thus contributing to existing research on this topic.

## Data Availability

The data of the studies is publicly available and could be accessible via website: (http://www.isss.edu.cn/cfps//EN/About/).

## Abbreviation

CFPS: China Family Panel Studies

## References

[1] WHO (2003). WHO Framework Convention on Tobacco Control.

[2] China Disease Control and Prevention Center. 2015 China Adult Tobacco Survey. [Chinese] Beijing. China: China CDC; 2015.

[3] Chen ZM, Peto R, Zhou M, Iona A, Smith M, Yang L (2015). Contrasting male and female trends in tobacco-attributed mortality in China: evidence from successive nationwide prospective cohort studies. Lancet..

[4] Cavelaars AE, Kunst AE, Geurts JJ, Crialesi R, Grötvedt L, Helmert U (2000). Educational differences in smoking: international comparison. Br Med J.

[5] Norman H, Mary S, Judith O, Gregory G (1991). Baseline factors associated with smoking cessation and relapse. Prev Med.

[6] Tillgren P, Haglund BJ, Lundberg M, Romelsjo A (1996). The sociodemographic pattern of tobacco cessation in the 1980s: results from a panel study ofliving condition surveys in Sweden. J Epidemiol Community Health.

[7] Waite LJ (1995). Does marriage matter?. Demography..

[8] Umberson D (1987). Family status and health behaviors: social control as a dimension of social integration. J Health Soc Behav.

[9] Chung W, Kim R (2015). Are married men healthier than single women? A Gender Comparison of the Health Effects of Marriage and Marital Satisfaction in East Asia. PloS One.

[10] Takagi D, Kondo N, Takada M, Hashimoto H (2014). Differences in spousal influence on smoking cessation by gender and education among Japanese couples. BMC Public Health.

[11] Hamadeh RR, Ahmed J, Al Kawari M, Bucheeri S (2018). Smoking behavior of males attending the quit tobacco clinics in Bahrain and their knowledge on tobacco smoking health hazards. BMC Public Health.

[12] Lin S-J (2010). Estimating the determinants of smoking behavior in Taiwan. Subst Use Misuse.

[13] Hao H (2016). Five amendments to population and family planning law. China Population Today.

[14] Basten S, Jiang Q (2014). China's family planning policies: recent reforms and future prospects. Stud Fam Plan.

[15] Zhao X, Zhou Y, Tan H, Lin H (2018). Spillover effects of children’s political status on elderly parents’ health in China. J Epidemiol Community Health.

[16] Li L, Wu X (2014). Housing price and entrepreneurship in China. J Comp Econ.

[17] Xie Y, Hu J (2014). An introduction to the China family panel studies (CFPS). Chin Sociol Rev.

[18] Walque DD (2007). Does education affect smoking behaviors? Evidence using the Vietnam draft as an instrument for college education. J Health Econ.

[19] Farrell P, Fuchs VR (1982). Schooling and health: the cigarette connection. J Health Econ.

[20] Grossman M (1972). On the concept of health capital and the demand for health. J Polit Econ.

[21] Liu S, Liang HY (2014). Study on the trend of age gap change of married couples and the reasons in China since 1990 [Chinese]. South China Population.

[22] Lin HX, Xiao D, Liu Z, Shi Q, Hajek P, Wang C (2019). National survey of smoking cessation provision in China. Tob Induc Dis.

[23] Lin HX, Lin Y, Zheng YT, Liu Z, Chang C. Design, development and randomised controlled trial of a smartphone application,‘QinTB’, for smoking cessation in tuberculosis patients: study protocol. BMJ Open. 2019;9(12):e031204. 10.1136/bmjopen-2019-031204 PMC7003393 31796480

[24] Brunet L, Pai M, Davids V, Ling G, Paradis G, Lenders R (2011). High prevalence of smoking among patients with suspected tuberculosis in South Africa. Eur Respir J.

